# Radiation pneumonitis prediction with dual-radiomics for esophageal cancer underwent radiotherapy

**DOI:** 10.1186/s13014-024-02462-1

**Published:** 2024-06-08

**Authors:** Chenyu Li, Ji Zhang, Boda Ning, Jiayi Xu, Zhixi Lin, Jicheng Zhang, Ninghang Tan, Xianwen Yu, Wanyu Su, Weihua Ni, Wenliang Yu, Jianping Wu, Guoquan Cao, Zhuo Cao, Congying Xie, Xiance Jin

**Affiliations:** 1 Radiotherapy Center, First Affiliated Hospital of Wenzhou Medical University, Wenzhou, 325000 China; 2 Department of Radiation Oncology, The Quzhou Affiliated Hospital of Wenzhou Medical University, Quzhou People’ s Hospital, Quzhou, 324000 China; 3 Department of Respiratory, Lishui People’s Hospital, Lishui, 323000 China; 4 School of Basic Medical Science, Wenzhou Medical University, Wenzhou, 325000 China; 5 Radiological Department, First Affiliated Hospital of Wenzhou Medical University, Wenzhou, 325000 China; 6 Cixi Biomedical Research Institute, Wenzhou Medical University, Zhejiang, 315000 China

**Keywords:** Esophageal cancer, Radiation pneumonitis, Radiomics, Dosiomics

## Abstract

**Background:**

To integrate radiomics and dosiomics features from multiple regions in the radiation pneumonia (RP grade ≥ 2) prediction for esophageal cancer (EC) patients underwent radiotherapy (RT).

**Methods:**

Total of 143 EC patients in the authors’ hospital (training and internal validation: 70%:30%) and 32 EC patients from another hospital (external validation) underwent RT from 2015 to 2022 were retrospectively reviewed and analyzed. Patients were dichotomized as positive (RP+) or negative (RP-) according to CTCAE V5.0. Models with radiomics and dosiomics features extracted from single region of interest (ROI), multiple ROIs and combined models were constructed and evaluated. A nomogram integrating radiomics score (Rad_score), dosiomics score (Dos_score), clinical factors, dose-volume histogram (DVH) factors, and mean lung dose (MLD) was also constructed and validated.

**Results:**

Models with Rad_score_Lung&Overlap and Dos_score_Lung&Overlap achieved a better area under curve (AUC) of 0.818 and 0.844 in the external validation in comparison with radiomics and dosiomics models with features extracted from single ROI. Combining four radiomics and dosiomics models using support vector machine (SVM) improved the AUC to 0.854 in the external validation. Nomogram integrating Rad_score, and Dos_score with clinical factors, DVH factors, and MLD further improved the RP prediction AUC to 0.937 and 0.912 in the internal and external validation, respectively.

**Conclusion:**

CT-based RP prediction model integrating radiomics and dosiomics features from multiple ROIs outperformed those with features from a single ROI with increased reliability for EC patients who underwent RT.

**Supplementary Information:**

The online version contains supplementary material available at 10.1186/s13014-024-02462-1.

## Background

Esophageal cancer (EC) is one of the most malignant diseases with its incidence and mortality rates ranking seventh and sixth globally [1]. Radiotherapy (RT) is an important component of the standard treatment for EC [2]. Especially, with the advancement in RT planning and delivering techniques, such as intensity-modulated radiation therapy (IMRT), volumetric modulated arc therapy (VMAT), and proton therapy, etc., better dose coverage and normal tissue protection have been achieved with improved RT outcomes for EC [3]. However, due to the close geometric relationship between lung and para-esophageal tissue, radiation pneumonitis (RP) is one of the major dose-limiting factors and toxicities during thoracic RT for EC, which also seriously impacts the life quality and treatment outcomes [4]. Therefore, early detection and intervention of RP are imperative to maximize the therapeutic gain for EC patients.

Traditionally, clinical factors, such as tumor stage, smoking history, tuberculosis, asthma, other preexisting lung diseases, concurrent chemotherapy, etc., and dosimetric factors extracted from the dose-volume histogram (DVH), such as the relative volume of lung irradiated by a specific threshold dose (Vx) or/and mean lung dose (MLD), have been intensively investigated as the risk factors in the assessment and prediction of RP [5–7]. Although some factors and dosimetric metrics appeared promising, there is still no consensus on the comparative importance of these predictors [8]. With the development of quantitative analysis using image features, radiomics features extracted from images and dosiomics features extracted from dose distributions have been demonstrated to improve the performance of RP prediction for EC patients who underwent RT [9–11].

There was a study indicated that radiomics models with features extracted from multiple regions of interest (ROIs) improved the RP prediction in comparison with features from single whole-lung for lung cancer [12]. Previous studies also demonstrated that adding radiomics features from subregion improves the survival prediction accuracy for EC patients who underwent concurrent chemoradiotherapy [13]. Therefore, we hypothesized that radiomics and dosiomics features extracted from multiple regions would improve the RP prediction for EC patients. The purpose of this study is to investigate the performance of radiomics models, dosiomics models, and integrated models with features from multiple regions in the RP prediction for EC patients who underwent RT. An external validation was also conducted to reduce the false-positive rates and improve the reproducibility of these RP prediction models.

## Methods

### Patients

**Fig. 1:**
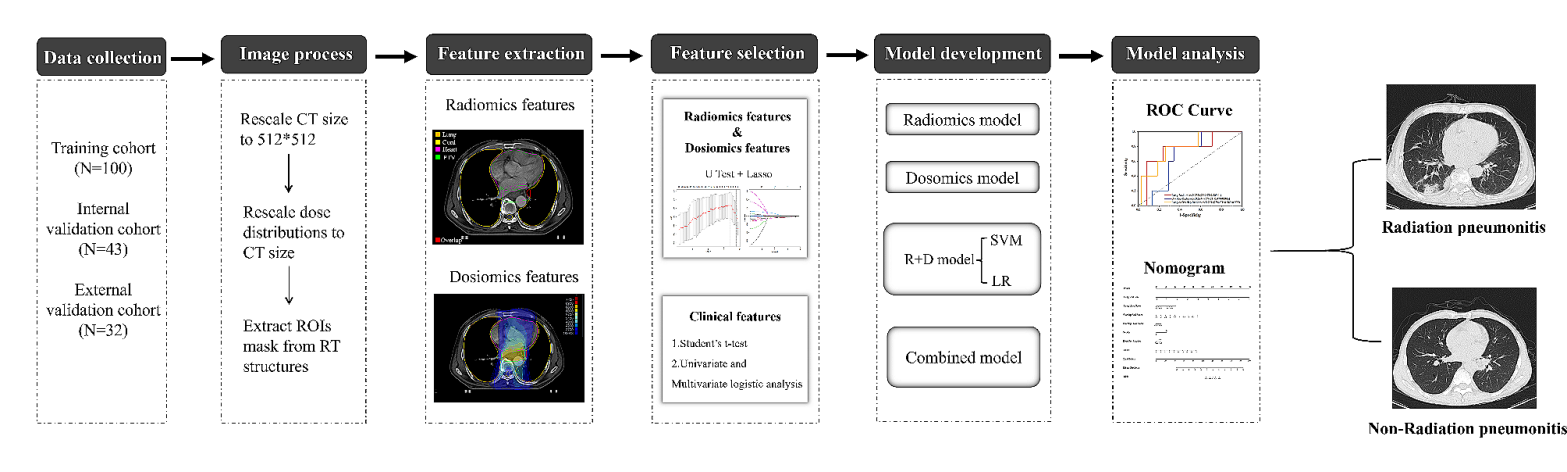
The flow diagram in the study

According to exclusion criteria (Appendices A), EC patients who underwent three-dimensional conformal RT (3D-CRT) or VMAT from 2015 to 2022 in the authors’ hospital were retrospectively reviewed and analyzed. Additional EC patients who underwent IMRT from another hospital with the same criteria were enrolled as an external validation cohort. The workflow for this study is shown in Fig. 1. The prescription doses for these EC patients were from 27 Gy to 64 Gy at 1.8 Gy to 3.0 Gy per fraction. During treatment planning with Pinnacle 9.2 (Philips Medical Systems, Andover, MA), the objective goal was to achieve 95% of the planning target volume (PTV) covered by 100% of the prescribed dose. Adaptive Convolve Algorithm was used to calculated the radiation dose with a dose gird of 4*4*4 mm. The dose limitations for organs at risk (OARs) were: maximum point dose of spinal cord < 45 Gy; lung V5 < 65%, lung V20 < 30%, lung V30 < 20%; heart V30 < 40%, etc. Detail beam setting and optimization parameters for 3DCRT, VMAT, and IMRT were reported previously [14, 15]. The Institutional Review Board of the authors’ hospital approved this retrospective study (IRB#2,019,059). The study was conducted according to the Declaration of Helsinki with the waived need for written informed consent due to its retrospective nature.

### Images and regions of interest

Planning computed tomography (pCT) of EC patients was acquired using a CT simulator with a 16-detector row (Brilliance, Phillips) under the same clinical protocol: 120 kV, 180–280 mA, and a field of view of 500 mm. An iodinated contrast of 100 mL at 300 mg/mL was injected intravenously before the CT scan. Images were reconstructed with a 3-mm section thickness for delineation of PTV and OARs by senior radiation oncologists. Images were resampled to a pixel spacing of 1 × 1 × 1 mm^3^ with B-spline interpolation algorithm to standardize feature computation [16]. The pixel values were distilled into equally spaced bins using a fixed bin width of 16 Hounsfield Units to eliminate the influence of different grayscale ranges and to ensure better comparability [17]. Radiomics and dosiomics features were extracted from two ROIs: the bilateral lung, and overlap volume, which was the overlapped volume between lung and PTV. Typical contours of PTV, OARs, lung, and overlapped volumes are shown in Appendices B.

### Feature extraction and selection

The radiomics features were extracted from each ROI using PyRadiomics [18]. The dose distributions were rescaled to the same size as CT images for dosiomics features extraction, in which the “image” consists of voxels with their grey level corresponding to the absolute dose in Gy [19]. A total of 1288 features were extracted including shape features, intensity features, and texture features, where the texture features were calculated from gray-level co-occurrence matrix (GLCM), ray-level run-length matrix (GLRLM), gray-level size zone matrix (GLSZM), gray-level dependence matrix (GLDM), and neighboring gray-tone difference matrix (NGTDM) [20]. The Mann-Whitney U test was first used to decrease the dimension of features with a *p* < 0.05 as potentially informative features in the prediction of RP, then the least absolute shrinkage and selection operator (LASSO) method with 5-fold cross-validation was applied to screen the optimal features by tuning the regulation weight λ to achieve a maximum area under the curve (AUC) of receiver operating characteristic (ROC) curves and to set the coefficients of irrelevant features exactly to zero [21].

### Model construction and validation

Patients from hospital one were divided into the training and internal validation sets at a ratio of 7:3. Models with radiomics (Rad_score) and dosiomics features (Dos_score) alone were constructed with the linear combination of selected features with their corresponding coefficients. Four models were constructed according to the source of features: Rad_score_Lung, Rad_score_Overlap; and Dos_score_Lung, Dos_score_Overlap. For the combining models with features from multiple ROIs, features extracted from lung and overlap regions were combined to go through the Mann-Whitney U test and LASSO for the final screen of optimal features, then models Rad_score_Lung & Overlap and Dos_score_Lung & Overlap were calculated based on the selected features and their corresponding coefficients. Logistic regression (LR) and support vector machine (SVM) were applied to construct combined radiomics and dosiomics models: Model A integrated Rad_score_Lung, Rad_score_Overlap, Dos_score_Lung and Dos_score_Overlap and Model B integrated Rad_score_Lung & Overlap and Dos_score_Lung & Overlap, respectively.

### Nomogram

Based on the univariate and multivariate analysis, clinical and DVH factors with *p* < 0.05 and MLD were included to construct a nomogram. A multivariable LR analysis was applied to build the radiomics and dosiomics-based nomogram integrating clinical and DVH factors. The performance of nomogram in both internal validation and external validation cohorts with calibration curves plotted using the Hosmer-Lemeshow (H-L) test. Decision curve analysis (DCA) in the internal validation and external validation dataset was plotted to evaluate the clinical value of the radiomics nomogram in this study [22].

### Follow-up and RP evaluation

After the treatment completion, EC Patients were followed up with a CT scan monthly in the first half year, and then every 3 months until 2 years. Immediate examination or intervention was administered for patients with symptoms, such as fever, cough, or shortness of breath during follow-up. RP was diagnosed by at least two radiation oncologists according to Common Terminology Criteria for Adverse Events (CTCAE) (Appendices G) [23]. Patients with obvious symptoms, indicated medical intervention, or had limiting instrumental activities of daily living were defined as positive RP+ (grade ≥ 2). Patients with less than grade 2 RP were defined as RP-.

### Statistical analysis

The clinical variables between the RP + and RP- groups were compared using the Fisher exact test or Chi-square test for categorical variables and the Mann-Whitney U test or independent-sample T-test for continuous variables. A two-tailed *p* value < 0.05 was defined as statistical significance. The statistical analysis was conducted with SPSS version 27.0 (IBM, Armonk, NY, USA). The LASSO and other statistical analyses were performed using the R analysis platform (version 5.0.1, MathSoft) along with the “glmnet” package (http://www.Rproject.org). The SVM model was performed using the “e1071” package with the confusion matrix performed using the “caret” package. The LR model was performed using the “glm” function and the “caret” package. The ROC curve was performed using the “pROC” package.

## Results

A total of 143 EC patients (123 male, 20 female) were recruited from hospital one as the training and internal validation sets, and another 32 patients (24 men, 8 women) from the second hospital were included as an external validation cohort with a mean age of 65 and 71, respectively. A total of 39 EC patients suffered from positive RP (RP+, 22.29%) with 26 (26%), 5 (11.63%), and 8 (25%) RP + patients in the training, internal validation, and external validation cohorts, respectively. There was a significant difference in gender between the RP + group and the RP- group in the training and internal validation cohorts, but not in the external validation cohort. MLD was statistically different between the RP + group and RP- group in the training cohort and external validation cohort, but not in the internal validation cohort. Table 1 presents a summary of the detailed patient characteristics.

**Table 1:** Characteristic of patients in the training, internal validation and external validation cohorts

Characteristic	Training cohort(n = 100)			Internal validation cohort(n = 43)			*p* Value	External validation cohort(n = 32)		
	RP-(n = 74)	RP+(n = 26)	*p* Value	RP-(n = 38)	RP+(n = 5)	*p* Value		RP-(n = 24)	RP+(n = 8)	*p* Value
Gender (N, %)			< 0.05*			0.01*	*0.99*			*0.64*
Female	6(8.11%)	8(31.77%)		3(7.89%)	3(60.00%)			17(70.83%)	1(12.50%)	
Male	68(91.89%)	18(69.23%)	^*a*^	35(92.11%)	2(40.00%)		^*a*^	7(29.17%)	7(87.50%)	^*d*^
Age(years)			*0.56*			0.01*	*0.21*			< 0.05*
Mean ± SD	65.76 ± 9.43	66.96 ± 7.77		62.61 ± 8.64	74.20 ± 11.43			70.17 ± 8.80	74.87 ± 3.94	
Range	46–87	53–79	^*b*^	42–80	64–89	^*b*^	^*b*^	54–82	70–80	^*c*^
Smoking (N, %)			*0.55*			*0.15*	*0.26*			*0.22*
Yes	32(43.24%)	13(50.00%)		15(39.47%)	0(0.00%)			14(58.33%)	2(25.00%)	
No	42(56.76%)	13(50.00%)	^*a*^	23(60.53%)	5(100.00%)	^*d*^	^*a*^	10(41.67%)	6(75.00%)	^*d*^
Total radiation dose (Gy)			*0.37*			*0.09*	*0.27*			*0.99*
Median	50	53		52	54			54	54	
Mean ± SD	50.09 ± 7.99	51.71 ± 7.60		52.09 ± 6.74	51.52 ± 4.57			53.60 ± 2.53	53.6 ± 2.99	
Range	27.00–64.00	30.00–60.00	^*b*^	40.00–60.00	45.00–56.00	^*c*^	^*b*^	50.00–60.00	50.96-60.00	^*c*^
Dose per fraction (Gy)			*0.99*			*0.24*	*0.45*			*0.68*
Median	1.8	2		2	1.8			1.8	1.81	
Mean ± SD	1.91 ± 0.21	1.92 ± 0.10		1.90 ± 0.12	1.84 ± 0.09			1.86 ± 1.53	1.83 ± .0.07	
Range	1.60-3.00	1.80-2.00	^*b*^	1.60–2.10	1.80-2.00	^*c*^	^*b*^	1.80–2.50	1.80-2.00	^*c*^
Chemotherapy history			*0.05*			*0.32*	*0.17*			*1.00*
Yes	44(59.46%)	21(80.77%)		28(73.68%)	5(100.00%)			17(70.83%)	6(75.00%)	
No	30(40.54%)	5(19.23%)	^*a*^	10(26.32%)	0(0.00%)	^*d*^	^*a*^	7(29.17%)	2(25.00%)	^*a*^
Chemoradiotherapy (N, %) Current Induction	34(77.27%) 10(22.73%)	15(71.43%) 6(28.57%)	*0.11* ^*d*^	23(82.14%) 5(17.86%)	3(60.00%) 2(40.00%)	*0.18* ^*d*^	*0.36* ^*a*^	12(70.59%) 5(29.41%)	4(66.67%) 2(33.33%)	*0.88* ^*d*^
Chemotherapy regimen (N, %)			0.03*			*0.15*	*0.17*			*0.59*
PF regimen	8(18.18%)	2(9.52%)		4(14.29%)	2(40.00%)			9(52.94%)	5(62.50%)	
TP regimen	36(81.82%)	19(90.48%)	^*d*^	24(85.71%)	3(60.00%)	^*d*^	^*d*^	8(29.41%)	3(37.50%)	^*d*^
MLD (Gy)			0.03*			*0.64*	*0.26*			0.03*
Median	10.63	11.17		12.63	10.30			11.69	9.17	
Mean ± SD	10.29 ± 4.03	11.84 ± 2.50		11.65 ± 4.01	10.78 ± 1.98			11.38 ± 2.26	9.18 ± 2.83	
Range	1.04–16.97	6.18–16.06	^*c*^	2.80-18.03	8.97–14.17	^*b*^	^*b*^	6.25–15.38	5.20-12.72	^*b*^

*Notes* RP + = RP grade ≥ 2; RP- = RP grade < 2; PTV = Planning Target Volume; Overlap = the overlap part of PTV and Lung; PF regimen: cisplatin + fluorouracil; TP regimen: paclitaxel + cisplatin; MLD = mean lung dose

^a^ Chi-squared test, ^b^ independent t test, ^c^ Mann-Whitney test, ^d^ Fisher’s precision probability test. * The *p* value < 0.05 is considered statistically significant

**Fig. 2:**
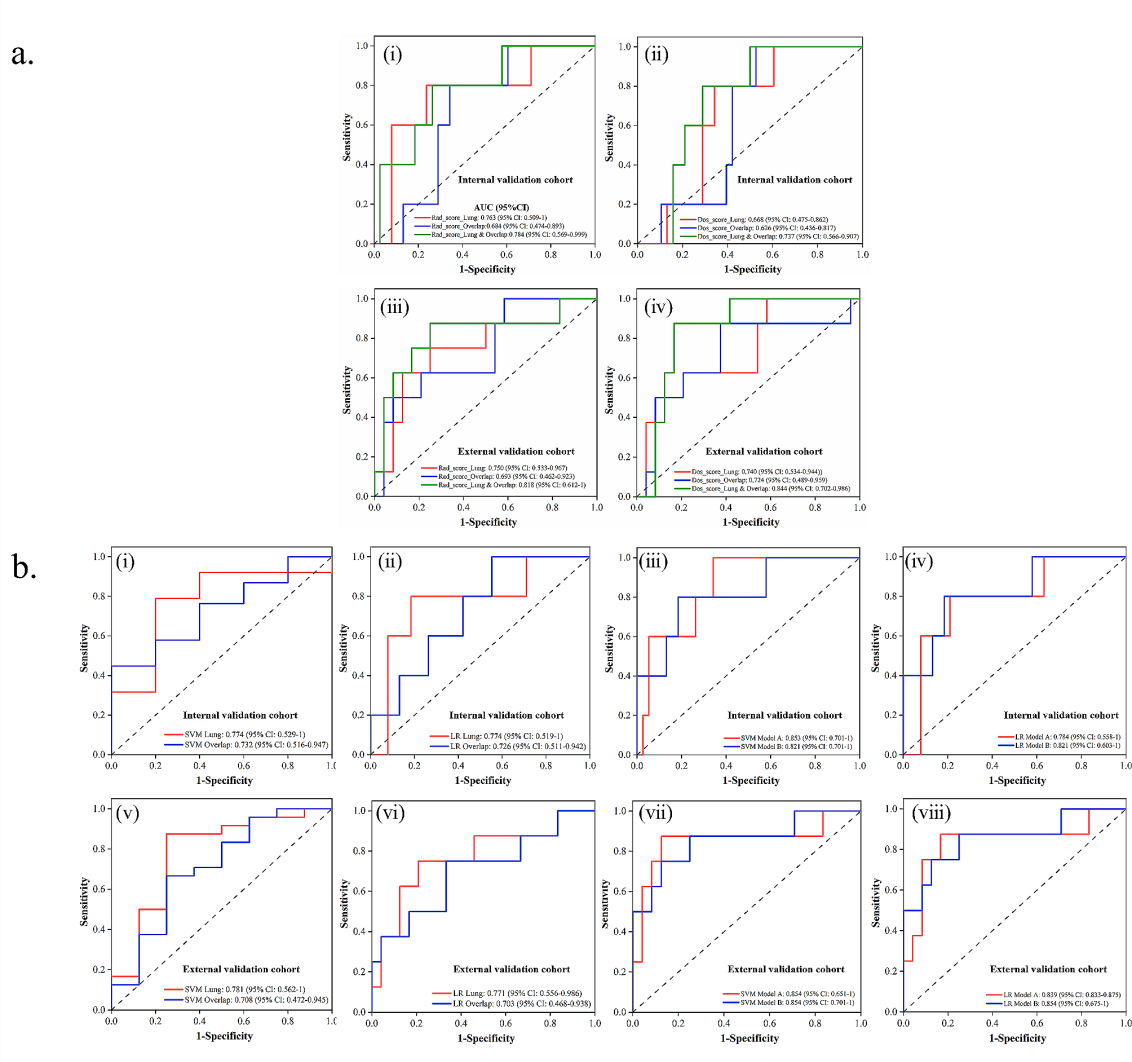
ROC curves of radiomics, dosiomics, radiomcis & dosiomics and machine learning models in validation cohort. The first row shows ROC curves of six models in the internal validation cohort (i)-(ii). The second row shows ROC curves of six models in the external validation cohort (iii)-(iv). The third row shows ROC curves of eight models in the internal validation cohort (i)-(iv). The fourth row shows ROC curves of eight models in the external validation cohort (v)-(viii)

There were 15 and 7 radiomics features, and 5 and 2 dosiomics features selected from Lung and Overlap regions, respectively. For multiple ROI models, there were 11 radiomics features (7 from Lung and 4 from Overlap) and 7 dosiomics features (5 from Lung and 2 from Overlap) were selected, respectively. The lists of these features along with their corresponding coefficients were shown in supplementary file Table D.1-D.4 and Fig. D.1-D.3. The risk score formulas were shown in supplementary file E. As shown in Fig. 2a, the AUC of Rad_score_Lung, Rad_score_Overlap, Dos_score_Lung, and Dos_score_Overlap was 0.763, 0.684, 0.668, 0.626; and 0.750, 0.693, 0.740, 0.724 in the internal and external validation, respectively. With multiple regions features, the AUC of Rad_score_Lung & Overlap and Dos_score_Lung & Overlap were 0.784 (95%CI, 0.569–0.999), 0.818 (95%CI, 0.569–0.999) and 0.737 (95%CI, 0.566–0.907), 0.844 (95%CI, 0.702–0.986) in the internal and external validation, respectively. Detailed performance of these models is shown in Table 2.

**Table 2:** The performance of the Radiomics and Dosiomics model for the training, internal validation and external validation cohorts

Model	Training cohort				Internal validation cohort				External validation cohort				
	AUC (95% CI)	Accuracy	Specificity	Sensitivity	AUC (95% CI)	Accuracy	Specificity	Sensitivity	AUC (95% CI)	Accuracy	Specificity	Sensitivity	
Rad_score_ Lung	0.898 (0.839–0.957)	0.780	0.703	1.000	0.763 (0.509-1)	0.786	0.763	0.800	0.750 (0.533–0.967)	0.750	0.750	0.750	
Rad_score_ Overlap	0.759 (0.651–0.867)	0.810	0.905	0.538	0.684 (0.474–0.893)	0.512	0.447	1.000	0.693 (0.462–0.923)	0.625	0.583	0.750	
Dos_score_ Lung	0.707 (0.591–0.823)	0.640	0.568	0.846	0.668 (0.475–0.862)	0.674	0.658	0.800	0.740 (0.534–0.944)	0.563	0.417	1.000	
Dos_score_ Overlap	0.720 (0.603–0.834)	0.750	0.784	0.654	0.626 (0.436–0.817)	0.534	0.474	1.000	0.724 (0.489–0.959)	0.688	0.625	0.875	
Rad_score_ Lung & Overlap	0.877 (0.810–0.944)	0.790	0.743	0.923	0.784 (0.569–0.999)	0.744	0.737	0.800	0.818 (0.612-1)	0.781	0.750	0.875	
Dos_score_ Lung & Overlap	0.781 (0.681–0.881)	0.720	0.703	0.769	0.737 (0.566–0.907)	0.674	0.711	0.800	0.844 (0.702–0.986)	0.844	0.833	0.875	

Models combining radiomics and dosiomics features using LR and SVM are shown in Fig. 2b and details are shown in Table 3. The AUC of Model A integrating Rad_score_Lung, Rad_score_Overlap, Dos_score_Lung, and Dos_score_Overlap was 0.853, 0.784, and 0.854, 0.839 in the internal and external validation cohorts using SVM and LR, respectively. Model B integrating Rad_socre_Lung & Overlap and Dos_score_Lung & Overlap achieved an AUC of 0.821, 0.821, and 0.854, 0.854 in the internal and external validation cohorts using SVM and LR, respectively.

**Table 3:** The performance of machine learning models for the training, internal validation and external validation cohorts

Model		Training cohort				Internal validation cohort				External validation cohort			
		AUC (95% CI)	Accuracy	Specificity	Sensitivity	AUC (95% CI)	Accuracy	Specificity	Sensitivity	AUC (95% CI)	Accuracy	Specificity	Sensitivity
SVM	Lung R + D	0.897 (0.835–0.959)	0.720	0.676	0.962	0.774 (0.529-1)	0.884	0.789	0.800	0.781 (0.562-1)	0.605	0.875	0.750
	Overlap R + D	0.787 (0.678–0.896)	0.680	0.703	0.808	0.732 (0.516–0.947)	0.605	0.447	1.000	0.708 (0.472–0.945)	0.512	0.667	0.750
	Model A	0.900 (0.832–0.967)	0.830	0.905	0.769	0.853 (0.701-1)	0.861	0.632	1.000	0.854 (0.651-1)	0.875	0.875	0.875
	Model B	0.890 (0.825–0.955)	0.730	0.824	0.846	0.821 (0.701-1)	0.465	0.816	0.800	0.854 (0.701-1)	0.813	0.875	0.750
LR	Lung R + D	0.904 (0.847–0.962)	0.780	0.703	1.000	0.774 (0.519-1)	0.814	0.816	0.800	0.771 (0.556–0.986)	0.781	0.792	0.750
	Overlap R + D	0.786 (0.676–0.895)	0.710	0.676	0.808	0.726 (0.511–0.942)	0.512	0.447	1.000	0.703 (0.468–0.938)	0.688	0.667	0.750
	Model A	0.908 (0.850–0.966)	0.740	0.649	1.000	0.784 (0.558-1)	0.791	0.789	0.800	0.839 (0.833–0.875)	0.843	0.833	0.875
	Model B	0.889 (0.823–0.954)	0.830	0.824	0.846	0.821 (0.603-1)	0.814	0.816	0.800	0.854 (0.675-1)	0.875	0.875	0.750

*Abbreviation* SVM = support vector machine; LR = logistic regression; Model A = Rad_score_Lung + Rad_score_Overlap + Dos_score_Lung + Doe_score_Overlap; Model B = Rad_score_Lung&Overlap + Dos_score_Lung&Overlap

According to the univariate analysis of clinical parameters (Supplementary file F), gender, age, smoking history, chemotherapy history, chemoradiotherapy, chemotherapy regimen, total radiation dose, dose per fraction, lung V5, lung V20, and lung V30 were the potential high-risk factors in the development of RP (all *p* < 0.05). Multivariate analysis revealed that gender (odds ratio [OR], 0.203; 95% CI, 0.057–0.718; *p* < 0.005) and lung V5 (OR: 0.958; 95% CI: 0.920–0.996) were independent predictors of RP. Prediction model with gender, lung V5 and MLD achieved an AUC of 0.684 and 0.858 in the internal validation cohort using SVM and LR, respectively. The details were shown in Supplementary file H.

**Fig. 3:**
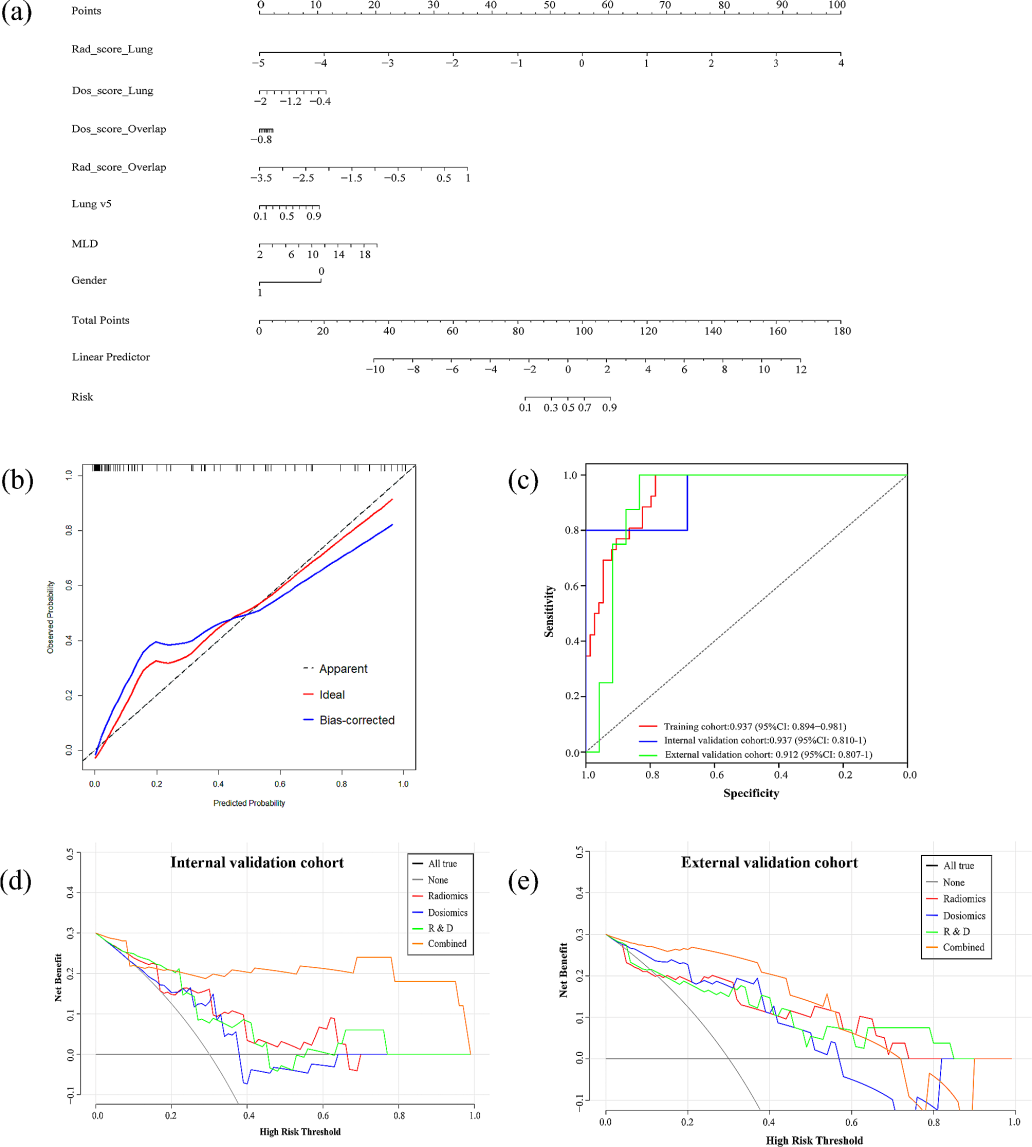
Nomogram of the combination models. (**a**) nomogram integrating Rad_score, Dos_score, clinical factors, DVH factors and MLD; (**b**) calibration curve of nomogram; (**c**) ROC curves of nomogram in the training cohort, internal validation cohort and external validation cohort; (**d**) DCA of internal validation cohort; (**e**) DCA of external validation cohort

Based on multivariate analysis, a nomogram (Fig. 3a) was conducted integrating Rad_score_Lung, Dos_score_Lung, Rad_score_Overlap, Dos_score_Overlap, gender, lung V5, and MLD. The calibration curve of the bootstrap resampling-validated nomogram is shown in Fig. 3b, which demonstrates a good agreement between the projected probabilities of RP and the true observed probabilities. The calibration curve resulting from the H-L test indicated an insignificant statistic in the internal validation (*p* = 0.512) and external validation (*p* = 0.619) cohorts. The AUC of the nomogram (Fig. 3c) was 0.937 and 0.912 in the internal and external validation cohorts, respectively. Figure 3d, e demonstrated the clinical viability and efficacy of the nomogram with decision curve analysis (DCA), which indicated that the integrated model with Rad_score, Dos_score, clinical parameters, DVH factors, and MLD showed the best positive net benefits at threshold probabilities.

## Discussion

In this study, radiomics and dosiomics features from multiple ROIs were integrated to predict the risk of RP for EC patients who underwent RT. Models with Rad_score_Lung & Overlap and Dos_score_Lung & Overlap achieved a better AUC of 0.818 and 0.844 in the external validation in comparison with radiomics and dosiomics models with features extracted from single ROI. Combining four radiomics and dosiomics models using SVM improved the AUC to 0.854 in the external validation. Nomogram integrating Rad_score, and Dos_score with clinical factors, DVH factors, and MLD further improved the RP prediction AUC to 0.937 and 0.912 in the internal and external validation, respectively.

The irradiation fields of RT for EC are usually large and complex to reduce the risk of recurrence in the subclinical region along the esophagus and regional lymph nodes [24]. Therefore, a healthy lung is inevitably irradiated to trigger the development of RP [25]. The incidence rate of RP + in this study was around 22%, which was similar to the reported 23.1% of RP with 3DCRT in the study of Lan et al. [26]. However, it was lower than the reported 34.95% RP ≥ 2 grade in the study of Du et al. [27]. Relatively lower RP rates were also reported in many other studies with a range from 15 to 40% [6]. These differences may result from different RT techniques applied. It may also be due to different adjuvant chemotherapy administered.

With the emergence of radiomics, studies demonstrated that it is promising for RP prediction for patients who underwent thoracic RT. In this study, Rad_score_Lung achieved an AUC of 0.763 and 0.750 with an accuracy of 0.786 and 0.750 in the internal and external validation, respectively. Similarly, Du et al. achieved an AUC of 0.765 in the independent validation cohort using cone beam CT in the prediction of RP for patients with esophageal squamous cell carcinoma (ESCC) undergoing RT [28]. Puttanawarut et al. also demonstrated that radiomics features from CT images achieved an AUC of 0.71 ± 0.10 in the RP prediction for EC patients who underwent RT [29]. In this study, the AUC of radiomics models was further increased to 0.784 and 0.818 with an accuracy of 0.744 and 0.781 in the internal and external validation cohorts, respectively, by combining features from lung and overlap regions (Rad_score_Lung & Overlap). To the best of our knowledge, few studies have reported radiomics features from multiple regions for RP prediction for EC patients. However, Kawahara et al. demonstrated that multi-region radiomics improved the AUC and accuracy from 0.62 to 60.8% to 0.84 and 80.1% in comparison with whole-lung radiomics in the RP prediction for patients with locally advanced non‑small cell lung cancer (NSCLC) treated by definitive RT [12].

In addition, previous studies demonstrated that DVH metrics are closely correlated with the reset of RP, but no universal parameters were accepted due to the heterogeneity across the studies [30, 31]. Consistently, the univariate analysis in this study demonstrated that all the selected DVH parameters and clinical factors were associated with RP of EC patients. Lung V5 was included in the nomogram in this study according to the multivariate analysis. And MLD was also considered as a factor of RP. This was consistent with previously reported findings [8, 31]. With the development of radiomics, dosiomics features have been regarded as containing profound information on DVH and dose distributions for RP prediction [11, 32, 33]. Puttanawarut et al. indicated that dosiomics features outperformed DVH parameters in the RP prediction for EC with an AUC of 0.71 [34]. Similarly, in this study, comparing to the model conducted by clinical and DVH parameters, Dos_score_Lung and Dos_score_Overlap achieved a higher AUC of 0.740 and 0.724 in the external validation, respectively. Dosiomics models with combined dosiomics features from multiple regions further improved the RP prediction with an AUC of 0.737 and 0.844 in the internal and external validation, respectively.

Combining radiomics and dosiomics features from the lung achieved an AUC of 0.774 and 0.781 in the internal and external validations, respectively. A similar AUC of 0.77 ± 0. 09 was reported in the study by Puttanawarut et al. in the RP prediction of EC by combining radiomics and dosiomics features [29]. Li et al. also reported an AUC of 0.849 ± 0.064 when combing radiomics and dosiomics from whole lung in the RP prediction for lung cancer patients [35]. In this study, combining radiomics and dosiomics features from multiple ROIs further improved the RP prediction, as shown in Table 3. SVM Model A integrating Rad_score_Lung, Rad_score_Overlap, Dos_score_Lung, and Dos_score_Overlap achieved a best AUC of 0.853 and 0.854 in the internal and external validation, respectively.

Wang L et al. achieved a C-index of 0.975 and 0.921 in the training and validation cohorts with a delta-radiomics nomogram, respectively, for the assessment of severe acute RP in EC with CT images following RT [10]. Lan K et al. developed a nomogram integrating non-smoking status, 3DCRT, lung V20, and PTV, for the prediction of symptomatic RP in ESCC patients received definitive concurrent chemoradiotherapy, and achieved an AUC of 0.772 and 0.900 in the primary and validation cohorts, respectively [36]. The nomogram in this study integrating Rad_score_Lung, Dos_score_Lung, Rad_score_Overlap, Dos_score_Overlap, gender, lung V5, and MLD further improved the AUC to 0.937 and 0.912 in the internal and external validation, respectively. The H-L test indicated that there was no significant deviation between the calibration curves and a perfect fit for predicting recurrence risk. The DCA results in Fig. 3d, e demonstrated the clinical viability and efficacy of the nomogram with best positive net benefits at threshold probabilities. This indicated that many patients could benefit from using the integrated RP prediction model to assist clinical decision-making.

One limitation of this retrospective study is the relatively small number of cases enrolled, even though external validation was conducted to strengthen the reproducibility of these models. Many risk factors have been reported to be associated with RP except for the dosimetric and clinical factors studied in this study, such as systemic therapies and intrinsic genetic phenotypes [37]. Integrating more related risk factors into these models will certainly further improve the prediction performance in the future. Another limitation is that the differences in data from different institutions were not considered thoroughly during data processing. In order to reduce the discrepancies caused by different data sources, appropriate data processing methods should be applied in future analyses. In this study, intersection of the lung and the PTV was treated as a sub-region, and exclude patients without overlap area between PTV and lung, which imposes limitations on the clinical application of the model.

## Conclusions

A CT-based RP prediction model integrating radiomics and dosiomics features from multiple ROIs was developed and validated externally for EC patients who underwent RT. Our findings demonstrated that models incorporating features from multiple ROIs outperformed those with features from a single ROI with increased reliability.

### Electronic supplementary material

Below is the link to the electronic supplementary material.

Supplementary Material 1

## Data Availability

The datasets used and/or analyzed during the current study are available from the corresponding author on reasonable request.

## Abbreviations

EC: Esophageal cancer
RT: Radiotherapy
IMRT: Intensity-modulated radiation therapy
VMAT: Volumetric modulated arc therapy
RP: Radiation pneumonitis
DVH: Dose-volume histogram
Vx: Specific threshold dose
MLD: Mean lung dose
ROIs: Regions of interest
3D-CRT: Three-dimensional conformal RT
PTV: Planning target volume
OARs: Organs at risk
pCT: Planning computed tomography
GLCM: Gray-level co-occurrence matrix
GLRLM: Ray-level run-length matrix
GLSZM: Gray-level size zone matrix
GLDM: Gray-level dependence matrix
NGTDM: Neighboring gray-tone difference matrix
LASSO: Least absolute shrinkage and selection operator
AUC: Area under the curve
ROC: Receiver operating characteristic
LR: Logistic regression
SVM: Support vector machine
H-L: Hosmer-Lemeshow
DCA: Decision curve analysis
CTCAE: Common Terminology Criteria for Adverse Events
