# Supplementary material for: Radiation pneumonitis prediction with dual-radiomics for esophageal cancer underwent radiotherapy
Source: Radiat Oncol. 2024 Jun 8;19:72. doi: 10.1186/s13014-024-02462-1 (PMC11161999; doi:10.1186/s13014-024-02462-1)
Supplement: Supplementary file 1 — Supplementary Material 1 [file 13014_2024_2462_MOESM1_ESM.docx]

**Supplementary Material**

1. **Exclusion criteria of patients**
2. **The contours PTV, OARs, lung, and overlapped volume**
3. **Radiomics and dosiomics features extraction**
4. **The details of the selected radiomics features and dosiomics features**
5. **Computational formula of Rad_score and Dos_score**
6. **Univariate and multivariate analysis of clinical factors in predicting RP**
7. **RP assessment**
8. **Discrimination ability of different models according to AUC with 95% CI**

**A.** **Exclusion criteria of patients**


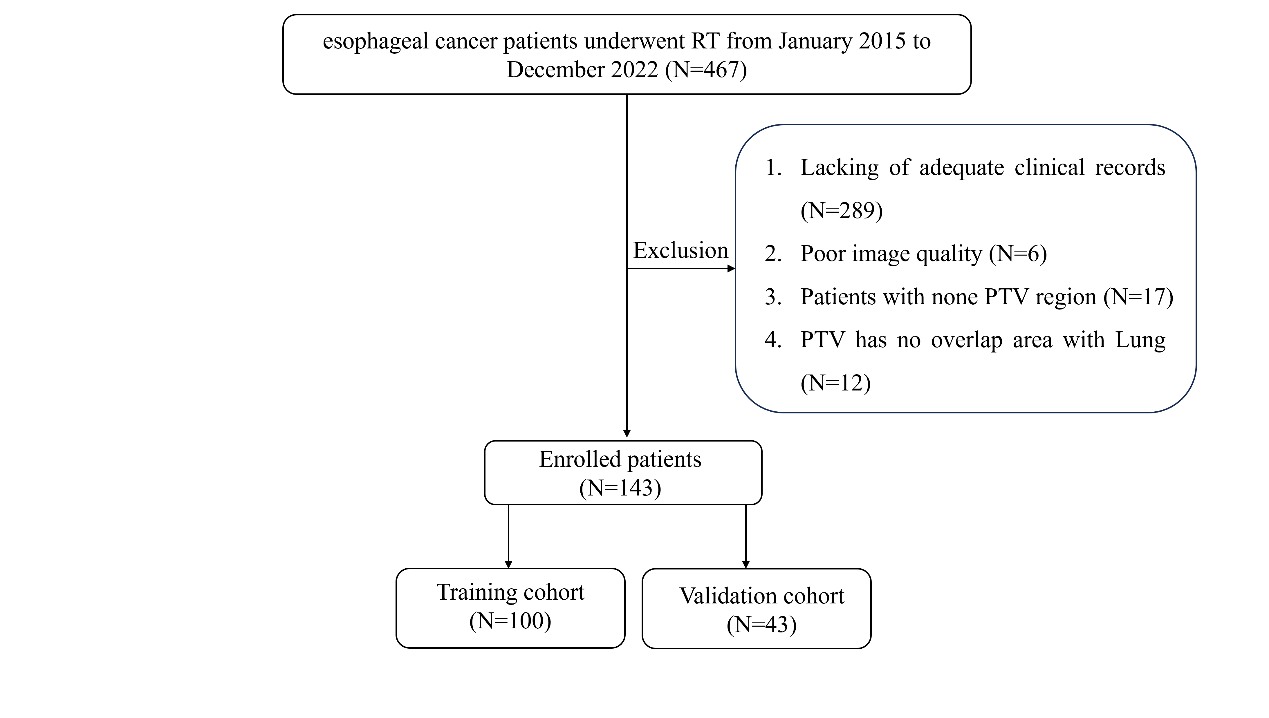


**B. The contours of PTV, OARs, lung, and overlapped volume**


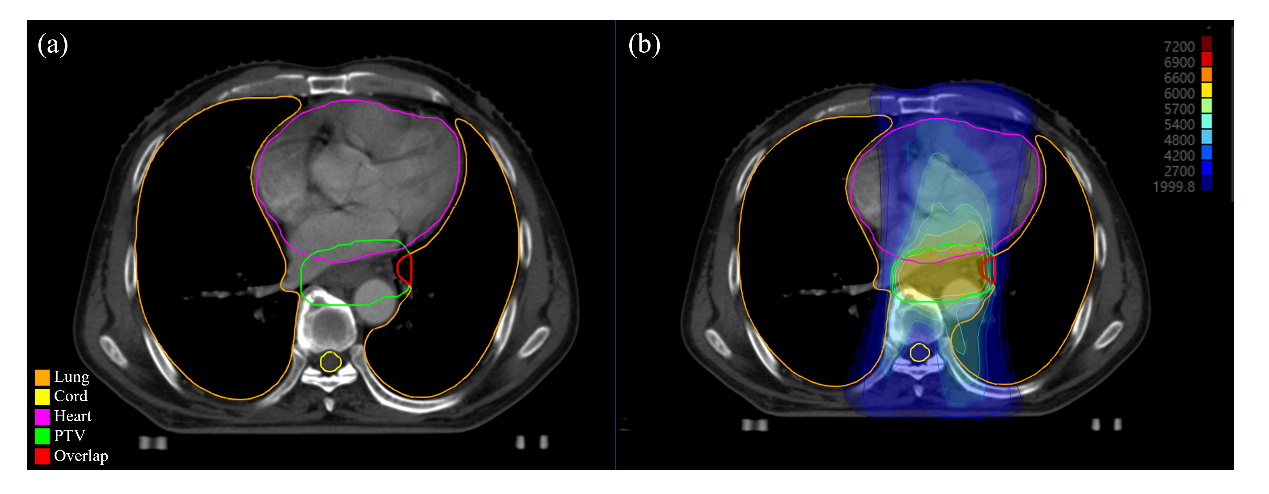


**(a) the contours; (b) dose distributions**

**C. Radiomics and dosiomics features extraction**

The same radiomics features and dosiomics features were extracted by Pyradiomics. Features are shown in the table below.

**Table.C.1 List of radiomics and dosiomics features extracted in this study.**

| Feature class | Features | Number of features |
| --- | --- | --- |
| Firstorder | 10Percentile,90Percentile,Energy,Entropy,InterquartileRange,Kurtosis,Maximum,MeanAbsoluteDeviation,Mean,Median,Minimum,Range,RobustMeanAbsoluteDeviation,RootMeanSquared,Skewness,TotalEnergy,Uniformity,Variance | 18 |
| Shape | Elongation,Flatness,LeastAxisLength,MajorAxisLength,Maximum2DDiameter(Column),Maximum2DDiameter(Row),Maximum2DDiameterSlice,Maximum3DDiameter,MeshVolume,MinorAxisLength,Sphericity,SurfaceArea,SurfaceVolumeRatio, VoxelVolume | 14 |
| GLCM | Autocorrelation,JointAverage,ClusterProminence,ClusterShade,ClusterTendency,Contrast,Correlation,DifferenceAverage,DifferenceEntropy,DifferenceVariance,JointEnergy, JointEntropy, Imc1, Imc2, Idm, Idmn, Id, Idn, InverseVariance, MaximumProbability, SumEntropy, SumSquares | 22 |
| GLSZM | GrayLevelNonUniformity,GrayLevelNonUniformityNormalized,GrayLevelVariance,HighGrayLevelZoneEmphasis,LargeAreaEmphasis,LargeAreaHighGrayLevelEmphasis,LargeAreaLowGrayLevelEmphasis,LowGrayLevelZoneEmphasis,SizeZoneNonUniformity,SizeZoneNonUniformityNormalized,SmallAreaEmphasis,SmallAreaHighGrayLevelEmphasis,SmallAreaLowGrayLevelEmphasis,ZoneEntropy,ZonePercentage,ZoneVariance | 16 |
| GLRLM | GrayLevelNonUniformity,GrayLevelNonUniformityNormalized,GrayLevelVariance,HighGrayLevelRunEmphasis,LongRunEmphasis,LongRunHighGrayLevelEmphasis,LongRunLowGrayLevelEmphasis,LowGrayLevelRunEmphasis,RunEntropy,RunLengthNonUniformity,RunLengthNonUniformityNormalized,RunPercentage,RunVariance,ShortRunEmphasis,ShortRunHighGrayLevelEmphasis, ShortRunLowGrayLevelEmphasis | 16 |
| NGTDM | Busyness,Coarseness,Complexity,Contrast,Strength, | 5 |
| GLDM | DependenceEntropy,DependenceNonUniformity,DependenceNonUniformityNormalized,DependenceVariance,GrayLevelNonUniformity,GrayLevelVariance,HighGrayLevelEmphasis,LargeDependenceEmphasis,LargeDependenceHighGrayLevelEmphasis,LargeDependenceLowGrayLevelEmphasis,LowGrayLevelEmphasis,SmallDependenceEmphasis,SmallDependenceHighGrayLevelEmphasis,SmallDependenceLowGrayLevelEmphasis | 14 |

**GLCM, gray-level co-occurrence matrix; GLRLM, gray-level run-length matrix; GLSZM, gray-level size zone matrix; NGTDM, neighborhood gray-tone difference matrix.**

**Note: it's worth noting that the names in this list are different from IBSI names, but the methods are the same. The algorithm details about feature extraction can be referred to:** [**https://pyradiomics.readthedocs.io**](https://pyradiomics.readthedocs.io)

Wavelet features were acquired using Coif wavelet on the basis of eight wavelet decompositions from three-dimensional sequences. All possible combinations of applying either a High or a Low pass filter include LLH, LHL, LHH, HLL, HLH, HHL, HHH and LLL. Therefore, 728 wavelet features were extracted.

The mathematical definitions are given on the Pyradiomics feature documentation (https://pyradiomics.readthedocs.io/en/latest/features.html). Most of them are described by the Imaging Biomarker Standardization Initiative (IBSI), which are available in a separate document by Zwanenburg et al (2016).

**D. The details of the selected radiomics features and dosiomics features**

**Table.D.1 The selected radiomics features based on CT.**

| ROI | Features |
| --- | --- |
| Lung | log-sigma-1-0-mm-3D_glszm_SmallAreaEmphasis  log-sigma-2-0-mm-3D_glszm_ZonePercentage  log-sigma-3-0-mm-3D_glszm_ZonePercentage  log-sigma-4-0-mm-3D_firstorder_Kurtosis  wavelet-LHL_firstorder_Skewness  wavelet-LHL_glcm_InverseVariance  wavelet-LHH_glcm_ClusterShade  wavelet-LHH_glcm_SumEntropy  wavelet-HLL_glszm_GrayLevelNonUniformityNormalized  wavelet-HLH_firstorder_Mean  wavelet-HLH_glrlm_LowGrayLevelRunEmphasis  wavelet-HLH_glszm_ZonePercentage  wavelet-HHH_glrlm_HighGrayLevelRunEmphasis  wavelet-HHH_glrlm_LowGrayLevelRunEmphasis  wavelet-HHH_glszm_SmallAreaLowGrayLevelEmphasis |
| Overlap | original_glcm_ClusterShade  log-sigma-2-0-mm-3D_firstorder_Skewness  wavelet-LHL_glcm_InverseVariance  wavelet-HLL_glcm_MaximumProbability  wavelet-HLH_firstorder_10Percentile  wavelet-HLH_glcm_ClusterShade  wavelet-HHL_gldm_DependenceVariance |

**Table.D.2 The selected dosiomics features based on 3D dose image.**

| ROI | Features |
| --- | --- |
| Lung | original_glcm_ClusterShade  original_glcm_ClusterTendency  log-sigma-1-0-mm-3D_glszm_SmallAreaLowGrayLevelEmphasis  log-sigma-1-0-mm-3D_ngtdm_Contrast  wavelet-LHL_ngtdm_Contrast |
| Overlap | log-sigma-4-0-mm-3D_gldm_DependenceVariance  wavelet-LHL_glszm_GrayLevelVariance |

**Table.D.3 The selected radiomics features based on the CT of multi-region model**

| ROI | Features |
| --- | --- |
| Lung | log-sigma-1-0-mm-3D_glszm_SmallAreaEmphasis  log-sigma-4-0-mm-3D_firstorder_Kurtosis  wavelet-LHL_firstorder_Skewness  wavelet-HLL_glszm_GrayLevelNonUniformityNormalized  wavelet-HLL_glszm_SmallAreaEmphasis  wavelet-HLH_firstorder_Mean  wavelet-HLH_glrlm_LowGrayLevelRunEmphasis |
| Overlap | log-sigma-2-0-mm-3D_firstorder_Skewness  wavelet-LHL_glcm_InverseVariance  wavelet-HLH_glcm_ClusterShade  wavelet-HHL_gldm_DependenceVariance |

**Table.D.4 The selected dosiomics features based on the 3D dose image of multi-region model**

| ROI | Features |
| --- | --- |
| Lung | original_glcm_ClusterShade  log-sigma-1-0-mm-3D_glszm_SmallAreaLowGrayLevelEmphasis  log-sigma-1-0-mm-3D_ngtdm_Contrast  wavelet-LHL_glrlm_GrayLevelNonUniformityNormalized  wavelet-LHL_ngtdm_Contrast |
| Overlap | log-sigma-5-0-mm-3D_gldm_DependenceVariance  wavelet-LHL_glszm_GrayLevelNonUniformityNormalized |

**Figure.D.1 Radiomics feature selection of inner dataset using the LASSO regression.**


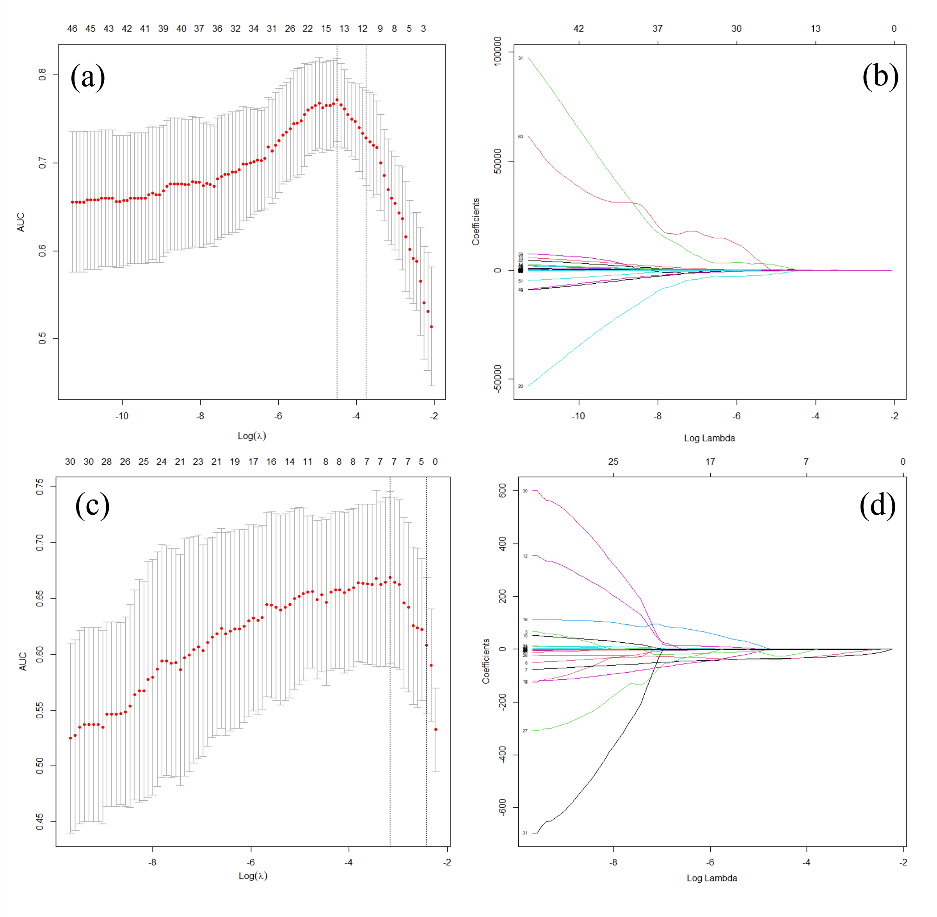


(a) The percent deviance of variable quantity explained and (b) Tuning parameter (λ) selection by the LASSO logistic model in the Lung radiomics features. (c) The percent deviance of variable quantity explained and (d) Tuning parameter (λ) selection by the LASSO logistic model in the Overlap radiomics features.

**Figure.D.2 Dosiomics feature selection using the LASSO regression.**


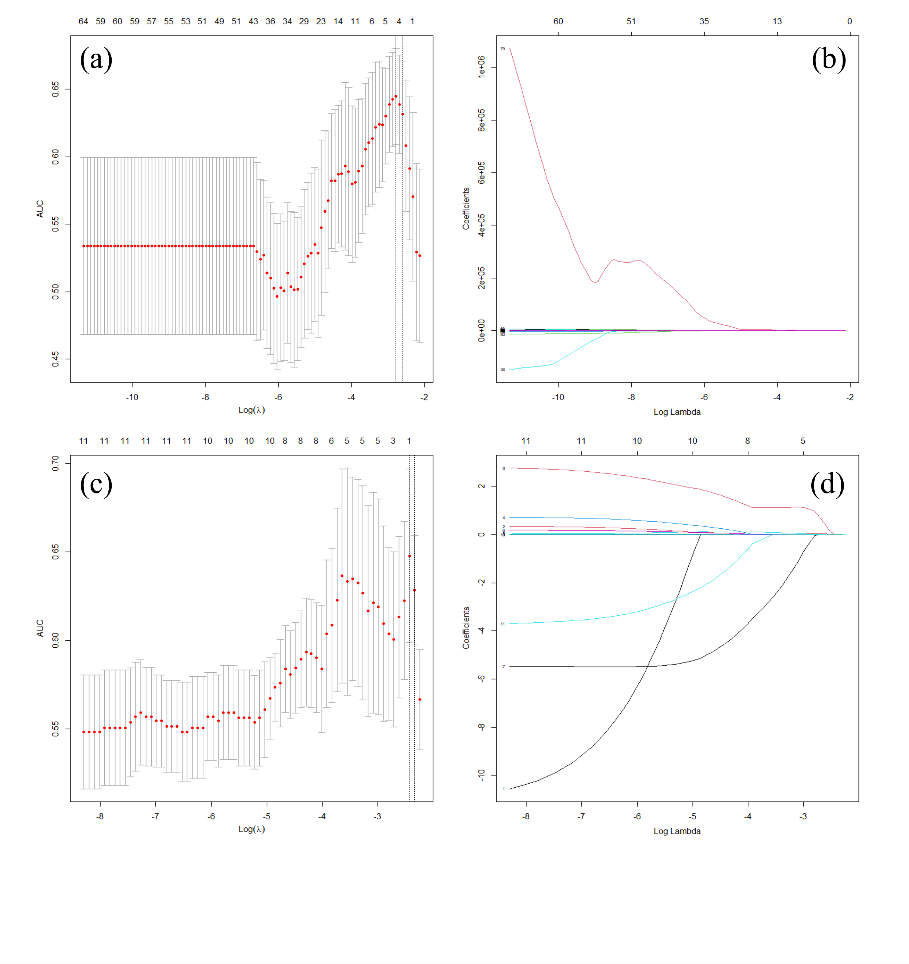


(a) The percent deviance of variable quantity explained and (b) Tuning parameter (λ) selection by the LASSO logistic model in the Lung dosiomics features. (c) The percent deviance of variable quantity explained and (d) Tuning parameter (λ) selection by the LASSO logistic model in the Overlap dosiomics features.

**Figure.D.3 the combination of radiomics features and dosiomics features selection using the LASSO regression.**


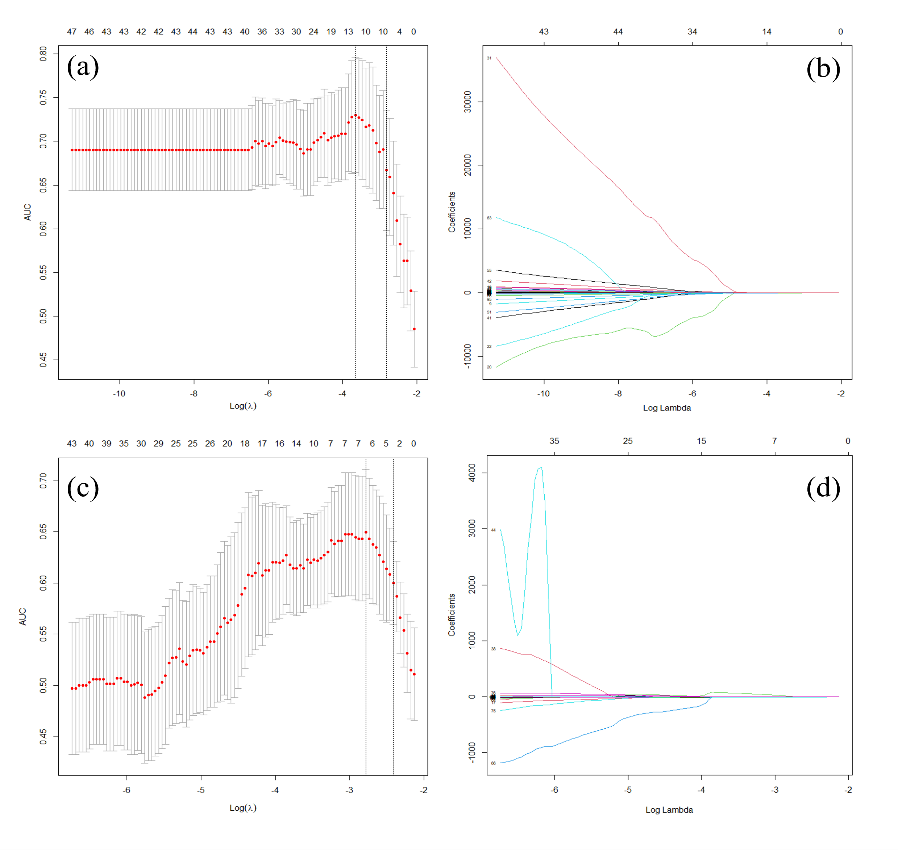


(a) The percent deviance of variable quantity explained and (b) Tuning parameter (λ) selection by the LASSO logistic model in the combination of radiomics features. (c) The percent deviance of variable quantity explained and (d) Tuning parameter (λ) selection by the LASSO logistic model in the combination of dosiomics features.

**E. Computational formula of radiomics score or dosiomics score**

**Eq. (E.1):** Rad_score_Lung formula:

$$-11.17092731+log-sigma-1-0-mm-3D\_glszm\_SmallAreaEmphasis*(9.602125318)+$$

$$log-sigma-2-0-mm-3D\_glszm\_ZonePercentage*(-363.8729281)+$$

$$log-sigma-3-0-mm-3D\_glszm\_ZonePercentage*\left( 161.7573443 \right)+$$

$$log-sigma-4-0-mm-3D\_firstorder\_Kurtosis*\left( -2.477386366 \right)+$$

$$wavelet-LHL\_firstorder\_Skewness*\left( 3.590786521 \right)+$$

$$wavelet-LHL\_glcm\_InverseVariance*\left( -26.16877634 \right)+$$

$$wavelet-LHH\_glcm\_ClusterShade*\left( -177.7809369 \right)+$$

$$wavelet-LHH\_glcm\_SumEntropy*\left( 12.85148087 \right)+$$

$$wavelet-HLL\_glszm\_GrayLevelNonUniformityNormalized*\left( -51.03105729 \right)+$$

$$wavelet-HLH\_firstorder\_Mean*\left( -1.729644242 \right)+$$

$$wavelet-HLH\_glrlm\_LowGrayLevelRunEmphasis*\left( 33.96318823 \right)+$$

$$wavelet-HLH\_glszm\_ZonePercentage*\left( 258.6099758 \right)+$$

$$wavelet-HHH\_glrlm\_HighGrayLevelRunEmphasis*\left( 1.720611558 \right)+$$

$$wavelet-HHH\_glrlm\_LowGrayLevelRunEmphasis*\left( -1.203487599 \right)+$$

$$wavelet-HHH\_glszm\_SmallAreaLowGrayLevelEmphasis*\left( 10.90177175 \right)+$$

**Eq. (E.2):** Dos_score_Lung formula:

$$-0.654079179+original\_glcm\_ClusterShade*(9.14E-08)+$$

$$original\_glcm\_ClusterTendency*(0.000527779)+$$

$$log-sigma-1-0-mm-3D\_glszm\_SmallAreaLowGrayLevelEmphasis*\left( -0.895894271 \right)+$$

$$log-sigma-1-0-mm-3D\_ngtdm\_Contrast*\left( -7.409392827 \right)+$$

$$wavelet-LHL\_ngtdm\_Contrast*\left( -8.936925416 \right)$$

**Eq. (E.3):** Rad_score_Overlap formula:

$$6.530799869+original\_glcm\_ClusterShade*(0.000461556)+$$

$$log-sigma-2-0-mm-3D\_firstorder\_Skewness*(-0.517062442)+$$

$$wavelet-LHL\_glcm\_InverseVariance*\left( -24.50309167 \right)+$$

$$wavelet-HLL\_glcm\_MaximumProbability*\left( -0.896843873 \right)+$$

$$wavelet-HLH\_firstorder\_10Percentile*\left( -0.26832796 \right)+$$

$$wavelet-HLH\_glcm\_ClusterShade*\left( -14.32925996 \right)+$$

$$wavelet-HHL\_gldm\_DependenceVariance*\left( 0.156920859 \right)$$

**Eq. (E.4):** Dos_score_Overlap formula:

$$-1.965532992+log-sigma-4-0-mm-3D\_gldm\_DependenceVariance*(0.027872086)+$$

$$wavelet-LHL\_glszm\_GrayLevelVariance*(0.135552025)$$

**Eq. (E.5):** Rad_score_Lung & Overlap formula:

$$7.156522396+lung-log-sigma-1-0-mm-3D\_glszm\_SmallAreaEmphasis*(14.25695884)+$$

$$lung-log-sigma-4-0-mm-3D\_firstorder\_Kurtosis*(-1.294918239)+$$

$$lung-wavelet-LHL\_firstorder\_Skewness*\left( 2.128575373 \right)+$$

$$lung-wavelet-HLL\_glszm\_GrayLevelNonUniformityNormalized*\left( -18.10486221 \right)+$$

$$lung-wavelet-HLL\_glszm\_SmallAreaEmphasis*\left( 3.900778485 \right)+$$

$$lung-wavelet-HLH\_firstorder\_Mean*\left( -36.11849018 \right)+$$

$$lung-wavelet-HLH\_glrlm\_LowGrayLevelRunEmphasis*\left( 8.10401064 \right)+$$

$$overlap-log-sigma-2-0-mm-3D\_firstorder\_Skewness*\left( -0.633940617 \right)+$$

$$overlap-wavelet-LHL\_glcm\_InverseVariance*\left( -30.46355508 \right)+$$

$$overlap-wavelet-HLH\_glcm\_ClusterShade*\left( -20.29350937 \right)+$$

$$overlap-wavelet-HHL\_gldm\_DependenceVariance*\left( 0.225997117 \right)$$

**Eq. (E.6):** Dos_score_Lung & Overlap formula:

$$-6.742869451+lung-original\_glcm\_ClusterShade*(6.62E-06)+$$

$$lung-log-sigma-1-0-mm-3D\_glszm\_SmallAreaLowGrayLevelEmphasis*(-1.151572647)+$$

$$lung-log-sigma-1-0-mm-3D\_ngtdm\_Contrast*\left( -5.804769799 \right)+$$

$$lung-wavelet-LHL\_glrlm\_GrayLevelNonUniformityNormalized*\left( 9.829271935 \right)+$$

$$lung-wavelet-LHL\_ngtdm\_Contrast*\left( -8.18682961 \right)+$$

$$overlap-log-sigma-5-0-mm-3D\_gldm\_DependenceVariance*\left( 0.047526981 \right)+$$

$$overlap-wavelet-LHL\_glszm\_GrayLevelNonUniformityNormalized*\left( -0.525038679 \right)$$

**F. Univariate and multivariate analysis of clinical factors in predicting RP**

| **Table. F1 Univariate and multivariate analysis of clinical factors in predicting RP** | | | | | | |
| --- | --- | --- | --- | --- | --- | --- |
| Characteristics | Univariate analysis | | | Multivariate analysis | | |
|  | *P* value | OR | 95% CI | *P* value | OR | 95% CI |
| Clinical factors |  |  |  |  |  |  |
| Gender | <0.001 | 0.265 | 0.157-0.445 | 0.013 | 0.203 | 0.057-0.718 |
| Age(years) | <0.001 | 0.985 | 0.978-0.991 | 0.644 | 1.012 | 0.961-1.066 |
| Smoking history | 0.006 | 0.406 | 0.213-0.774 | 0.268 | 1.804 | 0.635-5.125 |
| Chemotherapy history | <0.001 | 0.167 | 0.065-0.430 | 0.837 | 0.696 | 0.022-22.233 |
| Chemoradiotherapy | 0.005 | 0.659 | 0.492-0.882 | 0.654 | 0.734 | 0.191-2.831 |
| Chemotherapy regimen | 0.031 | 0.735 | 0.555-0.973 | 0.136 | 2.904 | 0.714-11.808 |
| Total radiation dose (Gy) | <0.001 | 1.000 | 1.000-1.000 | 0.376 | 1.000 | 1.000-1.001 |
| Dose per fraction (Gy) | <0.001 | 0.995 | 0.992-0.997 | 0.222 | 0.983 | 0.957-1.010 |
| DVH |  |  |  |  |  |  |
| Lung V5 | <0.001 | 0.981 | 0.973-0.989 | 0.032 | 0.958 | 0.920-0.996 |
| Lung V20 | <0.001 | 0.955 | 0.934-0.975 | 0.915 | 1.008 | 0.868-1.171 |
| Lung V30 | <0.001 | 0.929 | 0.894-0.964 | 0.255 | 1.108 | 0.929-1.321 |

Abbreviation: RP = radiation pneumonitis.

**G. RP assessment**

| Grade | Common Terminology Criteria for Adverse Events (CTCAE) v5.0 for RP |
| --- | --- |
| 0 | None |
| 1 | Asymptomatic; clinical or diagnostic observations only; intervention not indicated |
| 2 | Symptomatic; medical intervention indicated; limiting instrumental ADL |
| 3 | Severe symptoms; limiting self-care ADL; oxygen indicated |
| 4 | Life-threatening respiratory compromise; urgent intervention indicated (e.g., tracheotomy or intubation) |
| 5 | Death |

**H. Discrimination ability of different models according to AUC with 95% CI**

| Model | Training cohort | Internal validation cohort | External validation cohort |
| --- | --- | --- | --- |
|  | AUC (95%CI) | AUC (95%CI) | AUC (95%CI) |
| SVM |  |  |  |
| C | 0.668(0.537-0.800) | 0.684(0.332-1) | 0.661(0.417-0.906) |
| Rad_score+Dos_Score+C | 0.923(0.867-0.978) | 0.921(0.839-1) | 0.807(0.600-1) |
| LR |  |  |  |
| C | 0.650(0.516-0.784) | 0.658(0.293-1) | 0.380(0.122-0.639) |
| Rad_score+Dos_Score+C | 0.937(0.894-0.981) | 0.879(0.771-0.987) | 0.807(0.602-1) |

Note: C: gender + Lung V5 + MLD.
